# Supplementary figures and images for: Heat Shock Protein 27 Is Involved in the Bioactive Glass Induced Osteogenic Response of Human Mesenchymal Stem Cells
Source: Cells. 2023 Jan 5;12(2):224. doi: 10.3390/cells12020224 (PMC9856363; doi:10.3390/cells12020224)

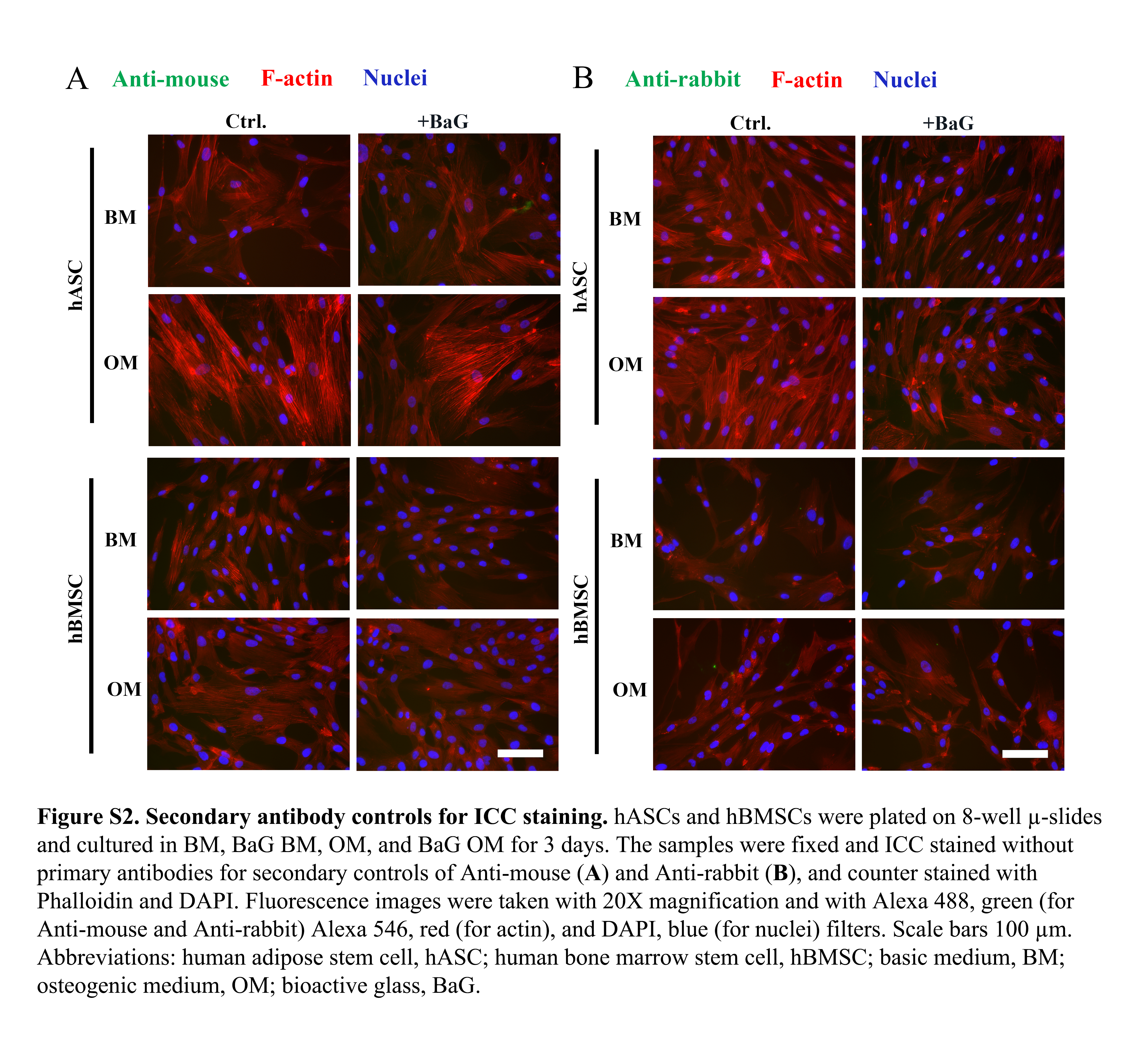

Supplement: Supplementary file 1 [file cells-12-00224-s001.zip › Figure S2.tif]

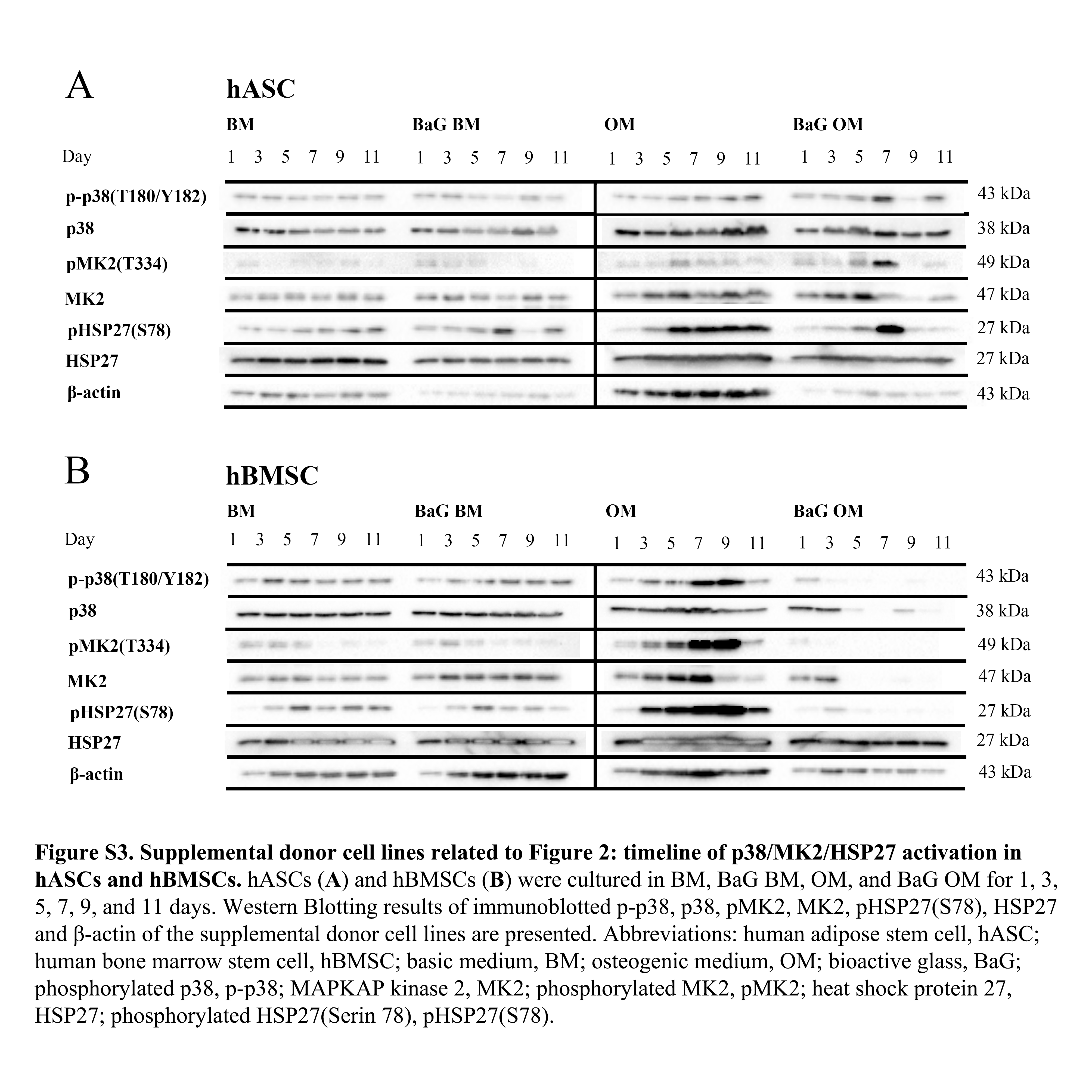

Supplement: Supplementary file 1 [file cells-12-00224-s001.zip › Figure S3.tif]

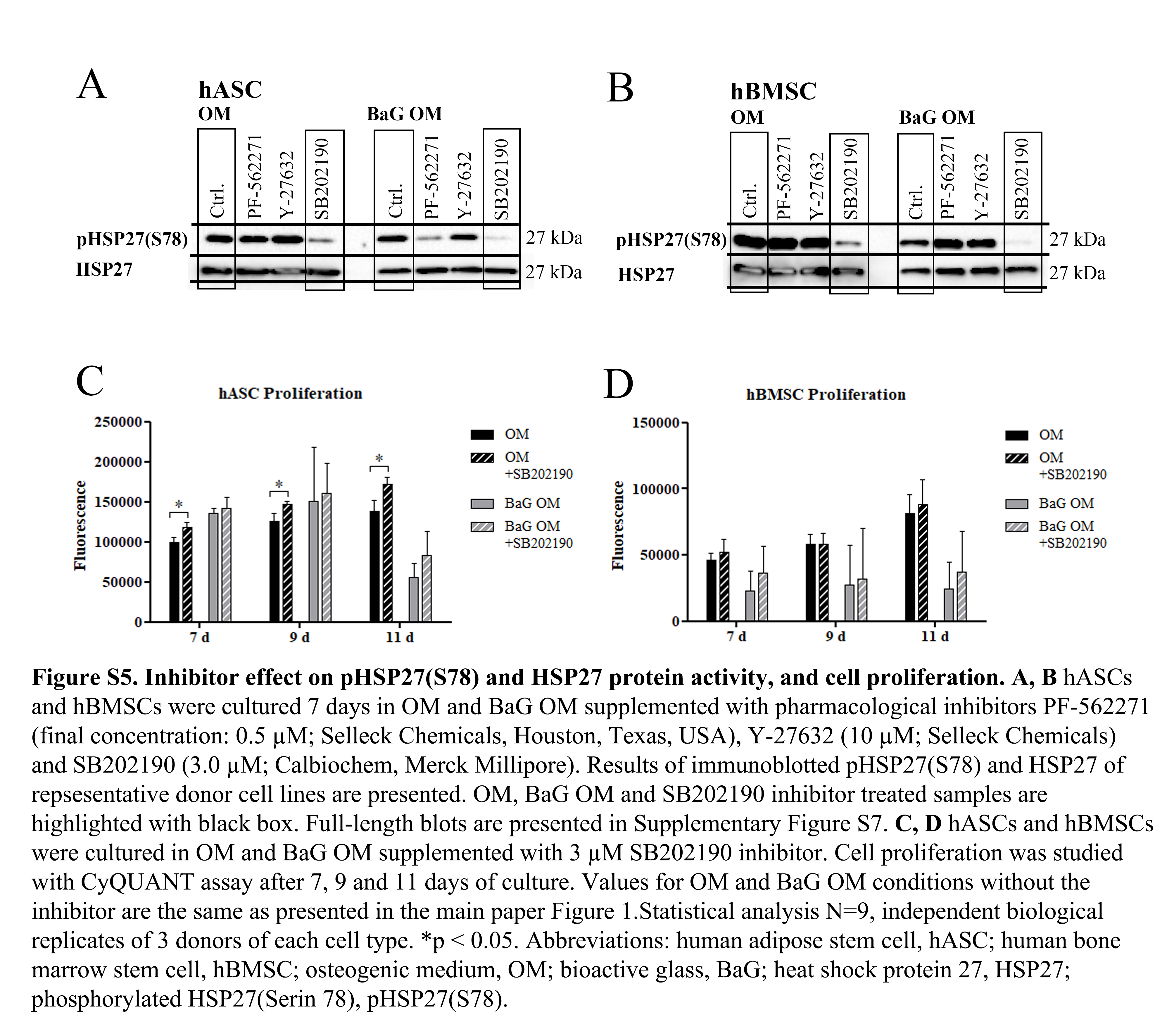

Supplement: Supplementary file 1 [file cells-12-00224-s001.zip › Figure S5.tif]

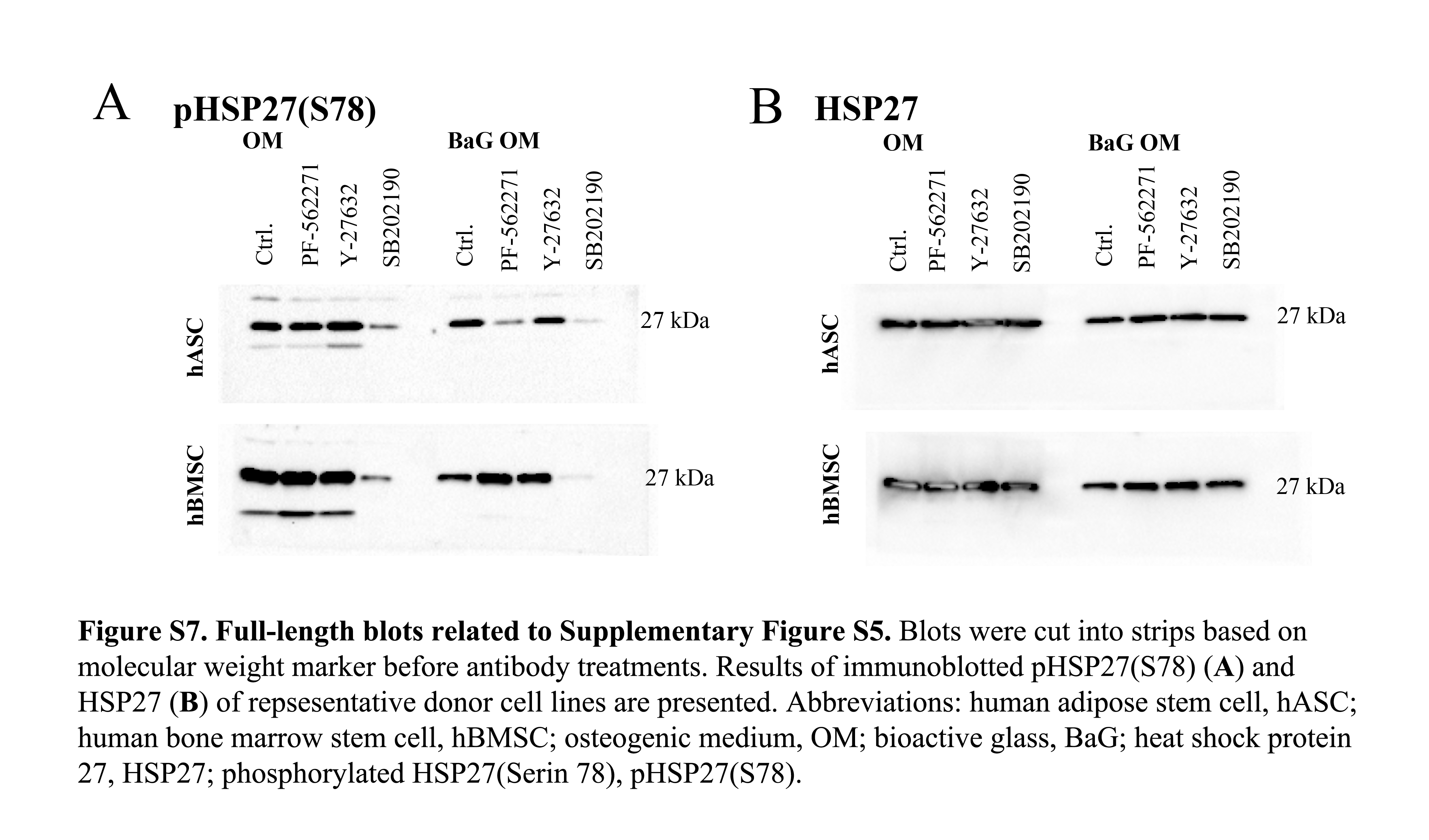

Supplement: Supplementary file 1 [file cells-12-00224-s001.zip › Figure S7.tif]
